# Exome sequencing study of partial agenesis of the corpus callosum in men with developmental delay, epilepsy, and microcephaly

**DOI:** 10.1002/mgg3.992

**Published:** 2019-10-02

**Authors:** Jolyane Meloche, Vanessa Brunet, Pierre‐Alexandre Gagnon, Marie‐Ève Lavoie, Jean‐Benoît Bouchard, Javad Nadaf, Jacek Majewski, Charles Morin, Catherine Laprise

**Affiliations:** ^1^ Centre intersectoriel en santé durable Université du Québec à Chicoutimi Saguenay QC Canada; ^2^ Département des Sciences Fondamentales Université du Québec à Chicoutimi Saguenay QC Canada; ^3^ Centre de Santé et de Services Sociaux de Chicoutimi Saguenay QC Canada; ^4^ Department of Human Genetics McGill University and Genome Quebec Innovation Centre Montreal QC Canada

**Keywords:** agenesis of the corpus callosum, *DCLK2*, exome sequencing, genetics

## Abstract

**Background:**

This study reports the genetic features of four Caucasian males from the Saguenay‒Lac‐St‐Jean region affected by partial agenesis of the corpus callosum (ACC) with hypotonia, epilepsy, developmental delay, microcephaly, hypoplasia, and autistic behavior.

**Methods:**

We performed whole exome sequencing (WES) to identify new genes involved in this pathological phenotype. The regions of interest were subsequently sequenced for family members.

**Results:**

Single‐nucleotide variations (SNVs) and insertions or deletions were detected in genes potentially implicated in brain defects observed in these patients. One patient did not have mutations in genes related to ACC, but carried a de novo pathogenic mutation in Mucolipin‐1 (*MCOLN1*) and was diagnosed with mucolipidosis type IV. Among the other probands, missense SNVs were observed in *DCLK2* (Doublecortin Like Kinase 2), *HERC2* (HECT And RLD Domain Containing E3 Ubiquitin Protein Ligase 2), and *KCNH3* (Potassium channel, voltage‐gated, subfamily H, member 3). One patient also carried a non‐frameshift insertion in *CACNA1A* (Cav2.1(P/Q‐type) calcium channels).

**Conclusion:**

Although no common genetic defect was observed in this study, we provide evidence for new avenues of investigation for ACC, such as molecular pathways involving *HERC2*, *CACNA1A*, *KCNH3,* and more importantly *DCLK2*. We also allowed to diagnose an individual with mucolipidosis type IV.

## Graphical abstract

We performed whole exome sequencing to identify new genes involved in partial agenesis of the corpus callosum with hypotonia, epilepsy, developmental delay, microcephaly, hypoplasia, and autistic behavior. Although no common genetic defect was observed, we provided new insights on genes potentially implicated in this pathological phenotype, such as *DCLK2*.

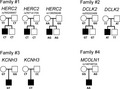

## 1 INTRODUCTION

The corpus callosum is the largest white matter tract in the human brain (Mihrshahi, 2006). It is essential for communication as it coordinates and transfers information between the two cerebral hemispheres (Aboitiz & Montiel, 2003). It plays a critical role in cognition, as well as in various sensory and motor functions (Mihrshahi, 2006). As early as 6 weeks of gestation, the 200 million axons that will become the corpus callosum are growing within the hemispheres. These fibers will start closing the gap between the 11th and 12th week of gestation. The partial or complete absence of this commissure is called a partial or complete agenesis of the corpus callosum (ACC). ACC is among the most frequent human brain malformations, occurring in 1:4000 individuals (Paul et al., 2007). Although the exact mechanisms implicated in ACC etiology are unknown, current evidence suggests that genetic alterations, such as single gene mutation or more complex genetic abnormalities, are implicated in its development.

The Saguenay–Lac‐St‐Jean (SLSJ) region is in the northeastern Quebec in Canada. Most of the SLSJ population is of French‐Canadian descent and this region was marked by three successive founder effects, which contributed to shape its unique genetic pool (De Braekeleer, Dallaire, & Mathieu, 1993). Because of this, several genetic disorders have been described in this population, such as the hereditary motor and sensory neuropathy associated with ACC, known as the Andermann syndrome.(OMIM #218000) This syndrome was described in 1972 in patients originating from the SLSJ and Charlevoix regions (Dupre et al., 2003; Larbrisseau, Vanasse, Brochu, & Jasmin, 1984). This disorder was classified as an autosomal recessive syndrome affecting the chromosomal region 15q13‐15 (*SLC12A6*, OMIM #604878) and characterized by ACC associated with a progressive motor neuropathy.

More recently, physicians noticed that some children affected by partial ACC in the SLSJ region had a specific phenotype. Their ACC was not associated with Andermann syndrome or other syndromes commonly associated with ACC (Bedeschi et al., 2006; Taylor & David, 1998; van Bon et al., 2008; Volpe et al., 2006). In contrast, they had a specific phenotype: partial ACC associated with microcephaly, hypoplasia, developmental delay, epilepsy, and autistic behavior. In this context, we hypothesized that there might be a common genetic disorder causing this pathological phenotype involving epilepsy, developmental delay, microcephaly, hypoplasia, as well as partial ACC. Consequently, the objective of this study was to define common genes and/or variations implicated in this pathological phenotype using whole exome sequencing (WES) on several individuals presenting these clinical symptoms. As shown by Topper et al., WES is a promising technique for identifying new genes involved in intellectual disability (Topper, Ober, & Das, 2011).

## 2 MATERIALS AND METHODS

### 2.1 Editorial policies and ethical considerations

Ethical approval was obtained from the appropriate institutional ethic committees (*Centre intégré universitaire de santé et de services sociaux* (CIUSSS) du SLSJ and *Université du Québec à Chicoutimi* (UQAC)) and all individuals gave written informed consent.

### 2.2 Sample selection

Following the genetic structure of the population of SLSJ, known for its founder effect (Scriver, 2001), we included patients with partial ACC associated with epilepsy (refractory seizures; mixed generalized or partial focal to bilateral tonic‐clonic seizures), delayed psychomotor development, microcephaly (head circumference at birth <3rd percentile), midfacial hypoplasia, low hair implantation, autism or autistic behavior, and with at least one grandparent native of SLSJ. These patients presented isolated partial ACC, as they did not have any other central nervous system disorders. At the neuropsychological level, all probands exhibited a profound intellectual retardation. These clinical characteristics will be considered as the “pathological phenotype.” The exclusion criteria were the presence of polyneuropathy and/or a chromosomal abnormality already documented. Medical files of all minors with these specific symptoms and their relatives were obtained from the participating medical center archives and were reviewed. A total of four patients with similar clinical characteristics were included in this study (Table 1). We also included parents of the patients with ACC, as well as their sibling (brothers in these cases), when possible, to compare genotypes. All probands were Caucasian males.

**Table 1:** Clinical and phenotypic data of the probands

Patient	Age	Clinical features shared by all probands (inclusion criteria)	Other diseases and medical condition
1	23	Partial agenesis of the corpus callosum Hypotonia Epilepsy Autistic behavior Delayed psychomotor development Midfacial hypoplasia Microcephaly Absence of polyneuropathy Absence of known chromosomal abnormalities	Attention deficit hyperactivity disorder Alternate esotropia (strabismus) Behavioral disorder Eczema Negative Angelman test Pityriasis rosea Gastritis
2	21		Hyperactivity Growth retardation at birth Delayed language
3	40		Anemia Major handicap (no words) Hypomagnesaemia Folic acid deficit Cerebral palsy Spastic quadriplegia Bowel obstruction Volvulus Scoliosis
4	18		Thoracic convexity to the right Fulminant hepatitis Asthma and food allergies Growth retardation at birth

### 2.3 Exome sequencing

DNA was extracted from blood samples of four probands and their relatives (parents, as well as brother when available) using the Blood & Cell Culture DNA Mini Kit (QIAGEN) according to the manufacturer's instructions. WES was performed on the four affected individuals at the McGill University and Genome Québec Innovation Center. Exome capture was performed with the SureSelect® High Throughput Library from Agilent (Agilent Technologies). Exon‐enriched DNA was then sequenced with the HiSeq2000 Illumina technology. Libraries were sequenced in paired‐end formats for read lengths of 100 base pairs. The sequencing reads were aligned to the NCBI human reference genome (NCBI, build GRCh37/hg19) using Burrows‐Wheeler Alignment tool (BWA) (Li & Durbin, 2009). Single‐nucleotide variations (SNVs) and small insertions and deletions (INDELs) were subsequently identified using VarScan. ANNOVAR (open bioinformatics) was used to classify and annotate variants (INDELs, SNVs). SIFT (Sorting Intolerant From Tolerant) and PolyPhen‐2 (Polymorphism Phenotyping v2) were used to assess the potential pathogenicity of nonsynonymous variants (Adzhubei et al., 2010; Ng & Henikoff, 2001). ExAC Browser was used to investigate the probability of loss of function intolerance for the candidate genes (Lek et al., 2016).

### 2.4 Bioinformatic filtering

Stringent thresholds were used for variant calling. Variants in sites covered at less than 10×, variants in sites covered in only one direction, as well as variants found in <4 reads or <5% of reads were excluded. Further variant filtering was performed: nongenic, intronic, and synonymous variants were excluded from our analyses. SNVs found in >1% controls when compared to the 1000 Genome Project, dbSNP, and the *Réseau de Médecine Génétique Appliquée* (Genome Quebec, Genome Canada) databases were excluded from this study. Among these, variants including splice variants, deletions, and truncating SNVs, that were predicted pathogenic (SIFT, PolyPhen‐2), were kept for analyses. Furthermore, to find a potential gene responsible for the pathogenic characteristics of these probands with ACC, we focused our research on genes involved in the development and/or the integrity of motoneurons based on a thorough search of the literature. Genes that were biologically relevant to the pathological phenotype were selected for the next steps.

### 2.5 Sanger sequencing

After narrowing our search to variants in genes known to play a role in the development or integrity of motoneurons, DNA sequencing for the region of interest in these genes was performed by Sanger sequencing at the *Plateforme de Séquençage et de Génotypage des Génomes* (*Centre Hospitalier Universitaire de Québec‐Université Laval, Québec,* Canada). This step was performed on DNA of the affected individual, as well as his parents and siblings (when possible) to confirm variants, and to identify their transmission patterns, or if they are de novo mutations.

## 3 RESULTS

To find impactful genes responsible for the pathogenic characteristics of these probands with partial ACC, WES analyses were performed and variations in genes involved in the development and/or the integrity of motoneurons were prioritized. With this stringent filtering process, no variation was common to all probands. Nevertheless, variations in four genes with potential biological implication on the development and/or integrity of motoneurons were identified in three of the four affected men (Table 2). Indeed, variations in *DCLK2* (Doublecortin Like Kinase 2, OMIM #613166), *HERC2* (HECT And RLD Domain Containing E3 Ubiquitin Protein Ligase 2, OMIM #605837), *KCNH3* (Potassium channel, voltage‐gated, subfamily H, member 3, OMIM #604527), and *CACNA1A* (calcium voltage‐gated channel subunit alpha1 A, OMIM #601011) were observed. Furthermore, Sanger sequencing was performed on DNA from the probands' parents to distinguish the transmission pattern or whether it is a de novo mutation. DNA from healthy siblings, brothers in both cases, also underwent sequencing to investigate whether they inherited these variations. This helped in shedding light on the potential clinical impact of these variations.

**Table 2:** Mutations and polymorphisms resulting in amino acid changes in genes potentially implicated in agenesis of the corpus callosum in affected men

Patient	Gene	SNP ID	HGVS	Frequency (ExAC)	Variation	Consequence	Impact (PolyPhen‐2, SIFT)	Proband genotype	Maternal genotype	Paternal genotype	Sibling genotype
1	*MCOLN1*	rs73003348	NC_000019.9:g.7593048C>T	3.04E‐03	missense SNV	p.Thr261Met	Benign, tolerated	CT			
	*HERC2*	rs765206957	NC_000015.9:g.28380739T>C	4.94E‐05	missense SNV	p.Ile4039Val	Possibly damaging	CT	TT	CT	CT
		rs757141755	NC_000015.9:g.28391439C>T	2.47E‐05	missense SNV	p.Arg3651His	Probably damaging	CT	TT	CC	CT
		rs138059246	NC_000015.9:g.28459392G>A	8.57E‐04	missense SNV	p.Arg2129Cys	Probably damaging	AG	AA	GG	AG
2	*DCLK2*	rs200222469	NC_000004.11:g.151000277G>A NC_000004.11:g.151000277G>T	1.89E‐04	missense SNV	p.Gly33Val	Benign, tolerated	GT	GT	GG	GT
		NA	NC_000004.11:g.151170745T>A	NA	missense SNV	p.Met661Lys	Probably damaging	AT	TT	AT	TT
3	*CACNA1A*	NA	NA	NA	non‐frameshift insertion	p.Pro2312_ Gln2313ins	unknown	possible homozygous			
	*KCNH3*	NA	NC_000012.11:g.49936607G>T	NA	missense SNV	p.Lys188Asn	Possibly damaging	GT	GT	GG	
		NA	NC_000012.11:g.49936608C>T	NA	missense SNV	p.His189Tyr	Possibly damaging	CT	CT	CC	
4	*MCOLN1*	rs148748724	NC_000019.9:g.7591493G>A	2.48E‐05	splice donor	c.405+1G>A	Pathogenic	AA (de novo mutation)	GG	AG	

Abbreviations: *CACNA1A*, calcium voltage‐gated channel subunit alpha1 A; *DCLK2*, Doublecortin Like Kinase 2; ExAC, Exome Aggregation Consortium; *HERC2*, HECT And RLD Domain Containing E3 Ubiquitin Protein Ligase 2; HGVS, The Human Genome Variation Society Nomenclature; *KCNH3*, Potassium channel, voltage‐gated, subfamily H, member 3; *MCOLN1*, Mucolipin‐1; NA, data not available; PolyPhen‐2, Polymorphism Phenotyping v2; SIFT, Sorting Intolerant From Tolerant; SNP ID, single‐nucleotide polymorphism database identification; SNV, Single‐nucleotide variation.

**Figure 1:**
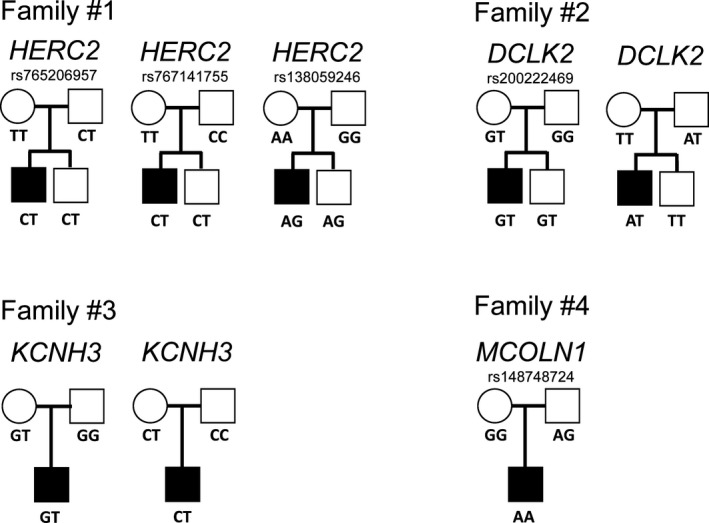
Genetic pedigrees of the four probands in this study. *DCLK2*, Doublecortin‐Like Kinase 2; *HERC2*, HECT And RLD Domain Containing E3 Ubiquitin Protein Ligase 2; *KCNH3*, Potassium channel, voltage‐gated, subfamily H, member 3; *MCOLN1*, Mucolipin‐1

Variations in *HERC2* (coding for HECT And RLD Domain Containing E3 Ubiquitin Protein Ligase 2), which is often associated with neurodevelopmental disorders, (Cubillos‐Rojas et al., 2016; Puffenberger et al., 2012; Tan, Bird, Thibert, & Williams, 2014) are relevant for one case (patient 1). This proband is heterozygous for three SNVs in this gene (rs765206957 (NC_000015.9:g.28380739T>C), rs757141755 (NC_000015.9:g.28391439C>T), rs138059246 (NC_000015.9:g.28459392G>A)), which were inherited from either one of his parents (Table 2). Thus, this proband has three multiple heterozygous variations in the *HERC2*, which may impact *HERC2* expression and protein production. Using PolyPhen‐2, the possible impact of amino acid substitution on protein function was predicted (Adzhubei et al., 2010). The impact of nonsynonymous variations is based on sequence homology and physical properties of amino acids (Table 2). According to PolyPhen‐2, two of these three SNVs are thought to be probably damaging the protein function. Nevertheless, his mother is homozygous for both these variations. The proband's brother (healthy) carries the same genetic profile for *HERC2*, as both siblings are heterozygous for these three variations (Figure 1).

Our analyses also pointed out two variations in the *DCLK2* (coding for Doublecortin Like Kinase 2) in another proband (patient 2). A missense variation (rs200222469 (NC_000004.11:g.151000277G>A, NC_000004.11:g.151000277G>T)) was identified along with another nonsynonymous SNV in *DCLK2* in this patient. Very little information is known on this second SNV at position chr4:151170745 (p.Met661Lys). Interestingly, the proband is the only family member affected by both these variations (Table 2 and Figure 1).

Furthermore, variations in two other genes were observed in our probands with the pathological phenotype. Patient 3 holds a non‐frameshift insertion in the *CACNA1A*, encoding for calcium voltage‐gated channel subunit alpha1 A. He also carries two variations in *KCNH3*, which encodes for the potassium channel, voltage‐gated, subfamily H, member 3. Nevertheless, this proband inherited these *KCNH3* variations from his mother. Unfortunately, we were unable to determine the transmission pattern for the variation in *CACNA1A* since adequate PCR amplification for this gene was impossible due to the nucleotide sequence specificities and difficulties in performing the technique.

Although, one patient (patient 4) did not have mutations in genes related to ACC, this study showed that he carried a de novo pathogenic mutation in Mucolipin‐1 (*MCOLN1*). A Mucolipidosis type IV diagnostic was made by a clinician and subsequent genetic counseling and screening was offered to the paternal family of this proband since one disease‐associated allele, based on ClinVar (rs148748724 (NC_000019.9:g.7591493G>A)), was transmitted by his father (Table 2). Another patient (patient 1) also carried a variation in *MCOLN1*, but this nonsynonymous variation is potentially benign according to PolyPhen‐2 (Polymorphism Phenotyping v2) prediction tool (Adzhubei et al., 2010). Thus, sequencing was not performed for this region of the gene.

## 4 DISCUSSION

Clinical manifestations of ACC, a common abnormality in the brain structure, vary from asymptomatic to delayed development, hypotonia, epilepsy, and microcephaly. In the SLSJ region, several pediatricians noticed that patients with partial ACC without polyneuropathy had hypotonia and later presented epilepsy, developmental delay, microcephaly, midfacial hypoplasia, low hair implantation, and autistic behavior (Table 1). WES was performed on four probands with the pathological phenotype and rare variants (<1%) in genes involved in the development and/or the integrity of motoneurons were highlighted. Although we were not able to identify a novel susceptibility variant for ACC in this study, we provide evidence for new avenues of investigation, such as molecular pathways involving *HERC2*, *CACNA1A*, *KCNH3,* and more importantly *DCLK2*. Indeed, the four probands, although they exhibit similar clinical characteristics, do not share a common genetic disorder. It is also possible that a common mutation was not discovered using WES, since this technique only covers exons. These analyses allowed to identify that, in most cases, at least one parent is a heterozygous carrier for the variations found in their affected child (Figure 1). Thus, further studies and functional assays are required to clearly understand whether these genes play a role in the development of ACC.

The implication of *HERC2* might not be surprising because of its implication in developmental delay, autism spectrum disorder, as well as Angelman‐like features (Puffenberger et al., 2012; Tan et al., 2014). Indeed, both studies identified a missense mutation in *HERC2* (rs397518474). Nevertheless, this mutation was not observed in patient 1 (Table 2). In another study, mutations in *HERC2* were associated with three cases of absence of the posterior half of the corpus callosum (Harlalka et al., 2013). In our study, although the proband's brother carries the same genotype for the *HERC2*, reduced penetrance and variable expressivity may affect the pattern of inheritance and may contribute to explain why only one sibling is affected by the disease. Further investigation is mandatory to demonstrate the pathogenic implication of the mutations found in this patient.

*KCNH3* is mainly expressed in the brain and was previously associated with cognitive function (Miyake et al., 2009). These alterations in cognitive function were also observed in *KCNH3* heterozygous mutant mice. Taken together, these alterations may impact the development and/or progression of ACC and associated disorders observed in this proband. Nevertheless, the affected child shares these mutations with his mother. Thus, taken alone, these mutations do not seem to induce ACC since the mother is not affected by this disease. More studies will be needed to demystify the underlying mechanisms involved in the types of interaction that regulate the genetics of this complex disease since the mutations potentially implicated in ACC development in patients 1 and 3 do not show regular segregation patterns, as concordance between genotypes and phenotypes is not always present.

Our results also pointed out the potential implication of the calcium channel CACNA1A. In the literature, a missense mutation in *CACNA1A* was observed in a patient affected by encephalopathy with a thin corpus callosum (Hayashida et al., 2018). Although our proband was not affected by the same mutation, a non‐frameshift insertion may alter the calcium channel formation and impact the development of ACC. Moreover, alterations in another isoform of this calcium channel were associated with ACC in a study by Sajan et al. (2013). They reported that rare copy number variants in *CACNA1B* could be considered as genetic risk factors in ACC patients. Furthermore, Damaj et al. also demonstrated that some patients carrying *CACNA1A* mutations develop epilepsy, autism, and cognitive impairment (Damaj et al., 2015). Thus, functional studies should be conducted to pinpoint the role of CACNA1A in the pathophysiology of ACC.

Regarding the implication of *DCLK* mutations, mice studies exhibited a role for DCLK in cortical neuronal migration and commissure formation. Deuel et al. showed that *Dclk* mutant mice exhibit axonal defects, which affected the corpus callosum (Deuel et al., 2006). Patient 2 holds missense mutations in *DCLK*, which could impact the development and/or progression of ACC and related disorders in this affected patient. Interestingly, the rs200222469 variant was reported in a patient with ACC in Geno_2_MP, a database of rare variants from the University of Washington Center for Mendelian Genomics (UW‐CMG). Furthermore, this proband is the only family member affected by both these mutations. Thus, this compound heterozygosity could have had a detrimental effect on the corpus callosum and unfortunately lead to ACC development by altering this serine/threonine‐protein kinase.

In conclusion, although the probands exhibited the same pathological phenotype, they do not seem to be affected by a common genetic disorder, but rather a combination of several diseases or syndromes presenting common clinical signs. Although our findings do not suggest a common ACC susceptibility gene, they provide new insights into molecular pathways involving *HERC2*, *CACNA1A*, *KCNH3,* and more interestingly *DCLK2* that could possibly be implicated in ACC development since they are all key players in motoneurons development and integrity.

## CONFLICT OF INTEREST

None.
